# Supplementary figures and images for: The Comparative Genomics of Botryosphaeriaceae Suggests Gene Families of Botryosphaeria dothidea Related to Pathogenicity on Chinese Hickory Tree
Source: J Fungi (Basel). 2024 Apr 22;10(4):299. doi: 10.3390/jof10040299 (PMC11051394; doi:10.3390/jof10040299)

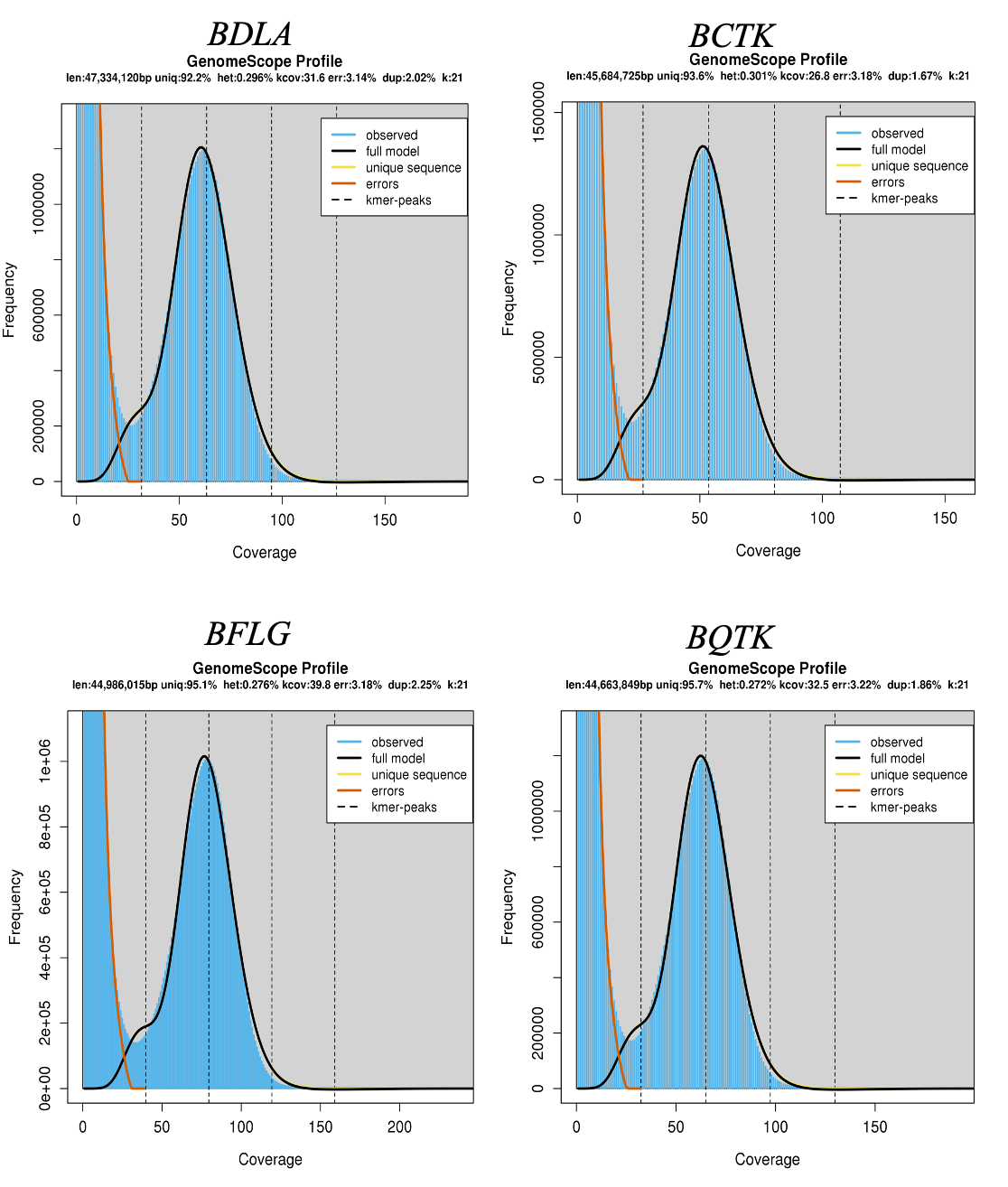

Supplement: Supplementary file 1 [file jof-10-00299-s001.zip › Figure S1.png]
